# Histology of the internal reproductive organs of the female Arabian oryx (*Oryx leucoryx*)

**DOI:** 10.1371/journal.pone.0308878

**Published:** 2024-10-24

**Authors:** Jose R. Rodriguez-Sosa, Jeffrey Plochocki, Saul Ruiz, Dominik Valdez, K. E. Beth Townsend

**Affiliations:** 1 Department of Anatomy, College of Graduate Studies, Midwestern University, Glendale, Arizona, United States of America; 2 College of Veterinary Medicine, Midwestern University, Glendale, Arizona, United States of America; 3 University of Central Florida, Orlando, Florida, Arizona, United States of America; Wrocław University of Environmental and Life Sciences: Uniwersytet Przyrodniczy we Wroclawiu, POLAND

## Abstract

The Arabian oryx (Oryx leucoryx) is an antelope that is considered a “vulnerable” species. Lack of knowledge on the structure of its reproductive organs hinders the development of strategies to improve its reproduction. There is only one report on the gross anatomy of the female reproductive organs. With the aim of describing their microanatomy, the ovaries, uterine tubes, uterus, and vagina were collected at necropsy of an adult female oryx. Samples from each organ were analyzed with H&E staining and light microscopy. The squamous to cuboidal germinal epithelium covers the dense tunica albuginea of the ovaries, which encloses the cortex. This merges smoothly with the medulla. The cortex embeds follicles at several stages of development. A rich bed of blood vessels is present in the medulla, especially at the hilum. The uterine tubes are comprised of a serosa, muscularis, and mucosa. The muscularis is constituted of circular smooth muscle covered by a thin layer of longitudinal fibers. The mucosa has longitudinal folds lined by a pseudostratified epithelium with ciliated cells. The uterine horns are constituted of a serosa, muscularis, and mucosa. The muscularis contains an inner circular layer of smooth muscle and an outer longitudinal one. Simple coiled glands are present at the base of the mucosa. From this, highly cellular projections (caruncles) are present, lined by a by a simple columnar epithelium. The cervix contains four dense rings, and its mucosa is lined by longitudinal folds of a simple columnar epithelium, with ciliated cells. Numerous glands are located in the lamina propria and contain simple cuboidal to columnar epithelium. The vaginal wall was made of a serosa, muscularis, and mucosa. The mucosa is made of stratified squamous epithelium non-keratinized. In summary, the histology of the internal reproductive organs of the oryx is like the one of other ruminants.

## Introduction

The Arabian oryx (*Oryx leucoryx*) is an antelope that belongs to the subfamily Hippotraginae of the family Bovidae. This species once inhabited the Sinai, lower Palestine, Transjordan, Iraq, and most of the Arabian Peninsula [1]. In 1930’s, the Arabian oryx began to be intensively hunted, and its remaining populations were found in the Northwest of Saudi Arabia. Overhunting eventually led to its extinction in the wild in 1972 [2]. Fortunately, in 1963, a few Arabian oryxes were captured and taken to the Arizona Center for Nature and Conservation/Phoenix Zoo, forming “The World Herd” [1]. Successful breeding led to the eventual reintroduction of the Arabian oryx [3] and the upgrade in the threat to extinction categories of the International Union for Conservation of Nature (IUCN) Red list, from “extinct in the wild” to “vulnerable”. According to the IUCN, the oryx wild population is stable or slowly growing. In 2016, this was composed of 1,220 animals, from which 850 were mature. Moreover, it is believed that approximately 6,000–7,000 individuals are held in captivity worldwide [4].

The Arabian oryx is well adapted to desertic conditions of the Arabian Peninsula [5], where it lives in herds of variable size [6]. The Arabian oryx reaches sexual maturity at 13 months and is polyestrous. Moreover, births can occur in all seasons, but peak during or after the rainy periods [7]. Its gestation length is ~255 days and, after calving, the females show their first estrous cycles at ~17 and 30 days in presence and absence of the male, respectively [8]. Observing the copulatory behavior, the length of the estrous cycle was estimated to be 19–29 days [7]. Using hormonal assays, the length of estrous cycle was determined to be 25–32 days [8].

Breeding in captivity has played a relevant role in the conservation of the Arabian oryx. Increasing its reproductive efficiency is essential if the status as a species is to be improved. The use of assisted reproductive technologies (ARTs), such as artificial insemination, embryo transfer, invitro fertilization, and gamete/embryo manipulation may help to reach that goal [9]. ARTs have been proven to be considerably useful in domestic animals, and important steps towards their application in the endangered mammalian species have been made [9, 10], including antelopes. For instance, the ultrasonographic-guided retrieval of oocytes and exogenous modulation of ovarian function have been reported in the eland (*Taurotragus oryx*) and bongo (*Tragelaphus eurycerus isaaci*) [11]. The artificial insemination the Indian blackbuck antelope (*Antilope cervicapra*) [12] and the production of embryos by intergeneric nuclear transfer of somatic cells in the bongo [13] and the Tibetan antelope (*Pantholops hodsomii*) [14–16] have been reported as well. In the Arabian oryx, the hormonal modulation of the ovarian function and embryo manipulation have been attempted, with mixed results [17–19]. However, the practical application of ARTs in the Arabian oryx and similar species is still a long way to go.

Deep understanding of the physiology and structure of the reproductive organs is essential for the artificial control of the reproductive function of any species. While the Arabian oryx was first named in 1777 and described in 1857 [6], the structure of its reproductive organs has been poorly studied. Lack of knowledge on their structural anatomy hinders the application of ARTs [9]. Regarding the Arabian oryx, in the best of our knowledge, only two reports are available on the structural aspects of these organs. They focus one the gross anatomy of the reproductive system in the male [20] and female [21]. The gross anatomy of the female reproductive organs is similar to that of domestic ruminants. However, the most notable difference is the absence of a distinctive uterine body and the presence of a cervix with two canals, one for each uterine horn that opens separately in each canal [21]. Because the microstructure of these organs had not yet been examined in this species, the objective of our study was to describe their histological features in the adult female Arabian oryx and contribute with this to the application of ARTs in this species.

## Material and methods

### Specimen

The ovaries, uterine tubes, uterine horns, cervix, and cranial region of the vagina were obtained from an adult (6-year-old) female Arabian oryx during its necropsy at the Arizona Center for Nature and Conservation/Phoenix Zoo. Her death was not due to any reproductive disease and there were no records indicating any abnormalities in her reproductive function. She was never exposed to a male and the hormonal modulation of its reproductive function was not attempted. The stage of the specimen was acceptable as the necropsy and collection of the organs were done immediately after death. Approval by an animal care and ethical committee was not required for this study due to it being based on analysis of tissue from a deceased animal. The specimen is located at Midwestern University as part of the Arizona Research Collection for Integrated Vertebrate Education and Study (ARCIVES) and cataloged as specimen ARC-M-068.

### Samples

The organs were measured using a vernier caliper and their size and gross anatomy were found to be like those reported in the normal female Arabian oryx (Table 1) [21]. Pieces of ~0.5 cm^3^ were obtained from each organ and immersed in 10% neutral buffered formalin. For the ovary, care was taken to bisect it through the longitudinal axis. Both halves were analyzed. The uterine tubes were dissected, extended, and cut transversally. A sample was taken from the ovarian, middle, and uterine regions. For the uterine horns, similar samples were obtained from the proximal, and distal regions. A sample was taken from the middle part of the cervix. Finally, a single sample was taken from the vagina.

**Table 1:** Dimension of internal reproductive organs of the oryx. The ones of the vagina are missing as the entire organ was not present.

Organ	Dimensions (cm)		
		Left	Right
Ovary			
	Length	1.8	1.6
	Width	1.5	1.6
	Height	1.2	1.0
Uterine tubes			
	Length	6.7	8.0
	Width	0.3	0.3
Uterine horns—Proximal			
	Length	12.3	14.0
	Width	1.0	1.3
Uterine horns—Distal			
	Width	1.5	1.9
Cervical canals			
	Length	3.1	3.0
	Width	1.6	1.7

### Histological analyses

Samples were processed, paraffin-embedded, cut (5 μm), mounted, stained with hematoxylin 560 (Leica Biosystems) and alcoholic eosin Y515 (Leica Biosystems) using a Leica ST5020 Stainer, coverslipped with a Leica CV5030 Coverslipper, and analyzed under light microscopy with an Olympus IX73 Inverted microscope. An overview of the section was initially obtained, and representative microphotographs were taken from the distinctive regions of the organs at several magnifications (x10—x100).

## Results and discussion

### Ovaries

**Fig 1:**
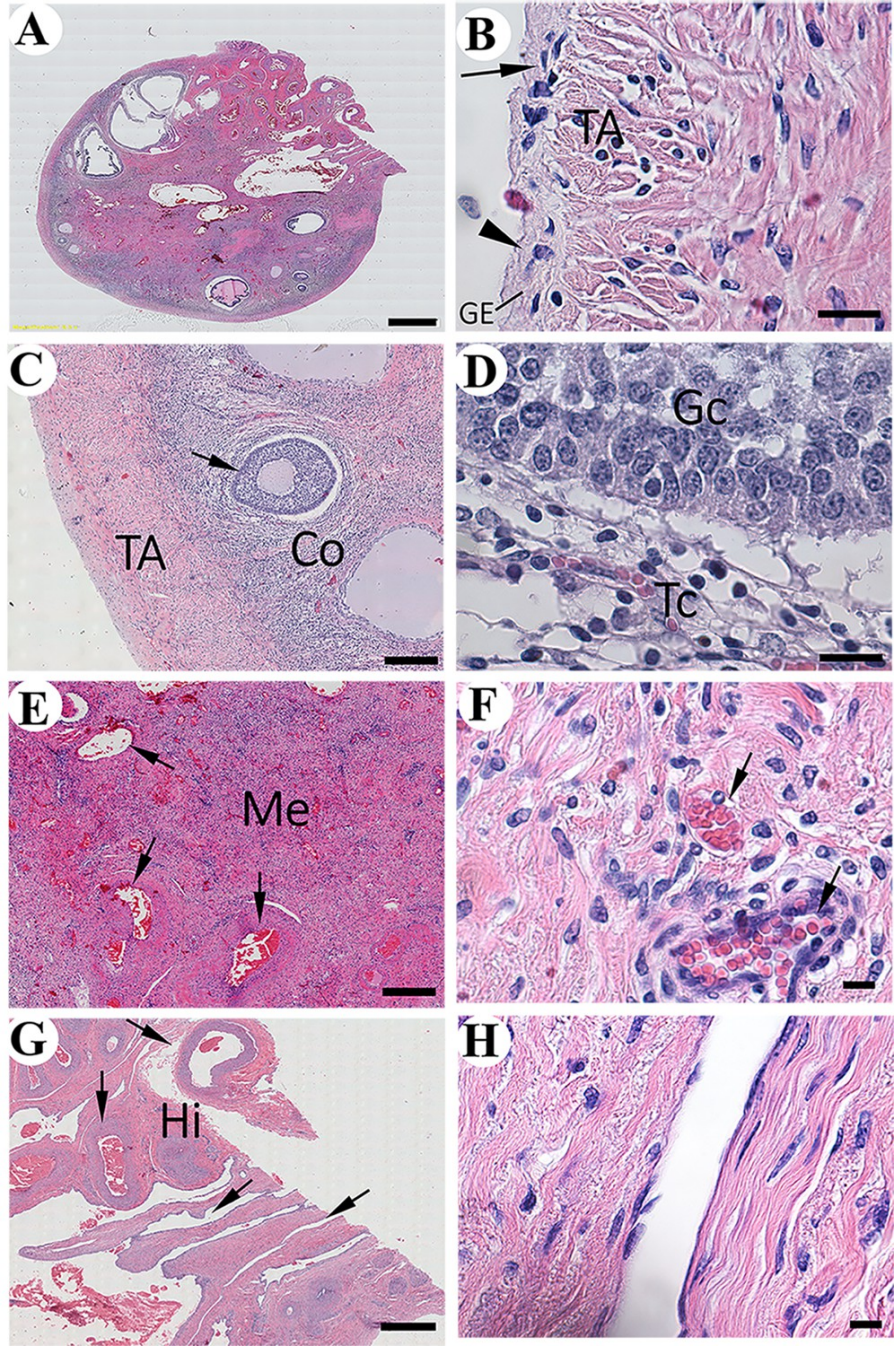
Histology of the ovary of the Arabian oryx. A) Overview of the longitudinal section of the right ovary (Bar = 2 mm). B) Tunica albuginea (TA). Simple squamous (arrow) and cuboidal cells (arrowhead) that constitute the germinal epithelium (GE) (Bar = 10 μm). C) Cortex (Co). Note the presence of antral follicles (arrow) (Bar = 250 μm). D) Wall of antral follicles with the Granulosa cells (Gc) surrounded by theca cells (Tc) (Bar = 20 μm). E) Medulla (Me) with large blood vessels (arrows) (Bar = 500 μm). F) Magnification of Me (20 μm). G) Hilum (Hi). Note the blood vessels (arrows) (Bar = 500 μm). H) Magnification of Hi showing the longitudinal section of a blood vessel (Bar = 20 μm).

The ovaries have the typical ovoidal shape (Fig 1A). At gross examination, several follicles in different stages of development were evident on their surface. The presence of antral follicles and absence of corpora lutea indicates that our specimen was in the initial stage of the estrous cycle or about to reach it [22]. The germinal epithelium, which covers the ovarian surface in other species, is present. It is a serous membrane homologous to the peritoneal layer of abdominal organs, including the reproductive ones. This layer is made of a simple squamous to cuboidal epithelium (Fig 1B), which is consistent with the description in the cow [23], and other species including human [24, 25]. The underlying tunica albuginea is evident supporting the germinal epithelium and is composed of fibrous connective tissue. The rest of the ovary shows distinctive cortical and medullary zones, which merges without a clear line of demarcation. The cortex stroma is made of a richly cellular connective tissue. It embeds blood vessels and follicles in different stages of development, including antral follicles (Fig 1C). This is in accordance to reports in Indian blackbuck antelope [26], giraffe (*Giraffa camelopardalis giraffa*) [27], cow [28], goat [29], ewe [30], and the mouse deer (*Tragulus javanicus* and *T*. *napu*) [31]. Antral follicles possess the typical organization described in cow [32] and other species [33]. They contain an oocyte, and the granulosa cells form a stratified columnar epithelium that is surrounded by the spindle-shaped theca cells (Fig 1D). Growth of follicles occurs in waves. The follicular waves in the estrous cycle of the Arabian oryx remain to be studied. However, considering the structural similarities with the Indian blackbuck antelope, cow, and ewes, the follicular waves of the Arabian oryx are probably like the ones of those species. In them, there are 2–3 follicular waves in the cycle [26, 28, 30]. Together with the ovarian follicles, numerous blood vessels are embedded by the cortical stroma. These extend from the medulla which merges without a clear line of demarcation and is the core of the organ (Fig 1E). It is made of abundant connective tissue embedding numerous blood vessels which extend from the hilum to the cortex (Fig 1F–1H). This is in accordance with what has been described in several species mentioned above [26–30, 33]. These blood vessels are likely derived from the *ramus tubaris* of the ovarian artery, like in the cow [34].

### Uterine tubes

**Fig 2:**
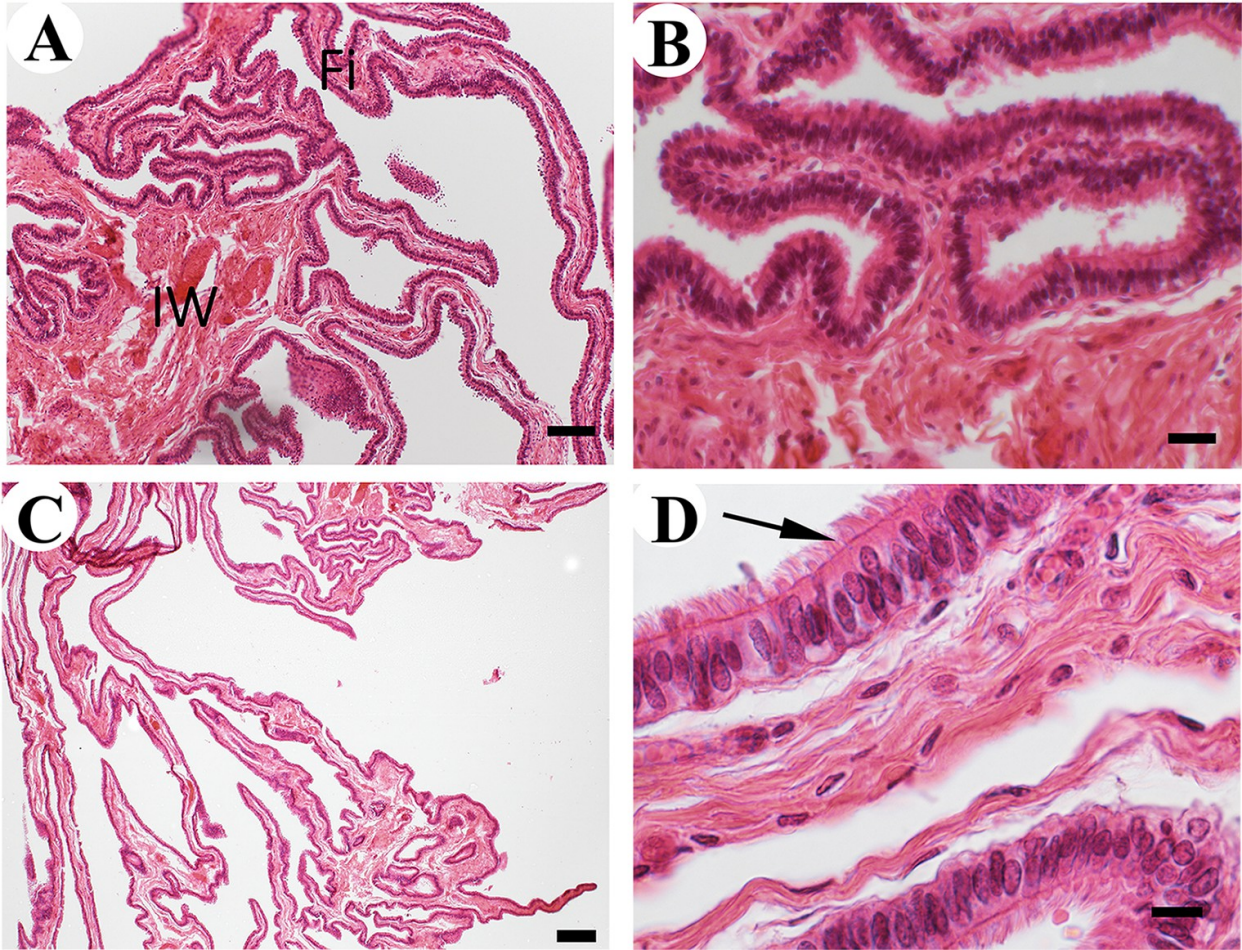
Histology of the infundibulum of the uterine tube of the Arabian oryx. A) Infundibular wall (IW). Note the base of the fimbria (Fi) (Bar = 100 μm). B) Base of the fimbria projections (Bar = 20 μm). C) Fimbria projections (Bar = 100 μm). D) Pseudostratified columnar epithelium with ciliated cells (arrow) (Bar = 10μm).

**Fig 3:**
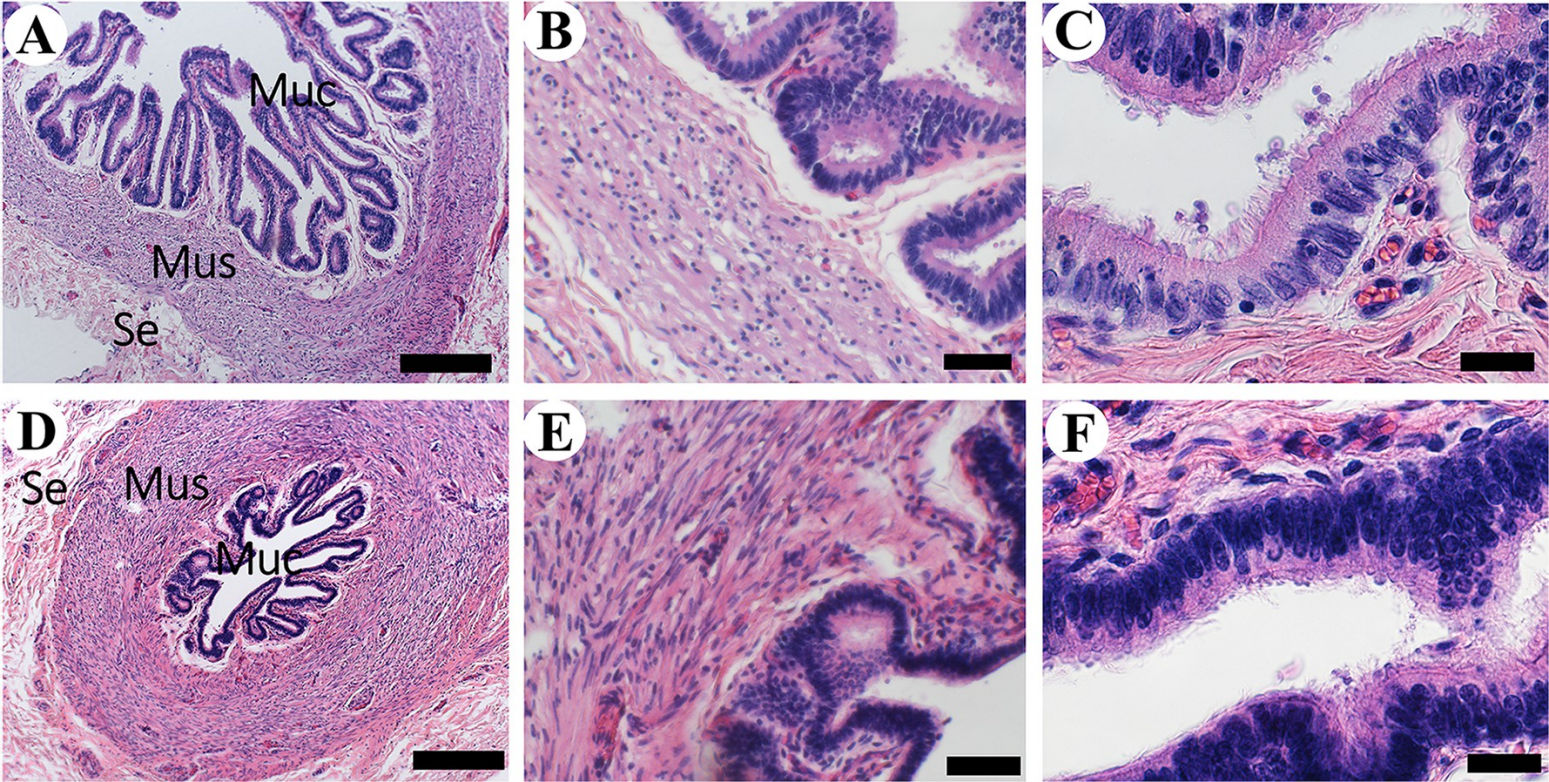
Histology of the ampulla and isthmus of the uterine tube of the Arabian oryx. A) Overview of the transversal region of the ampulla. Serosa (Se), muscularis (Mus), and mucosa (Muc) (Bar = 500 μm). B) Magnification of Mus and Muc (Bar = 50 μm) C) Lining of Muc, pseudostratified columnar epithelium (Bar = 20 μm). D) Overview of the transversal section of the isthmus (Bar = 500 μm). E) Magnification of Mus and Muc (Bar = 50 μm). F) Lining of Muc, with pseudostratified columnar epithelium (Bar = 20 μm).

The uterine tubes, formerly called oviducts, are the smallest organs of the internal reproductive tract. They are a pair of convoluted tubes associated with oocyte collection, fertilization, and early embryo development. Three regions can be distinguished along their length: infundibulum, ampulla, and isthmus [22]. In accordance with general descriptions [24] the elements that constitute the wall of these regions are similar along the length of the uterine tubes, albeit of some morphological differences. Their wall is made of the serosa, muscularis, and mucosa. The infundibulum is easily identified because of its funnel-like shape. Like in cow [22], the free border of this region possesses primary projections that tend to merge with each other (Fig 2A and 2B). A large muscularis layer is in the base of the primary folds, mostly made of fibers with a roughly oblique orientation. There is not clearly division into outer and inner layers. These primary projections extend farther to form the secondary long and thin ‘finger-like’ projections that constitute the fimbriae (Fig 2C), which is associated with collection of the oocytes after ovulation [22]. Like in the cow [35] and goat [36], the epithelium is made of ciliated and non-ciliated cells (Fig 2D). The uniform length of the cilia is consistent with our belief that our specimen was in the follicular phase of the estrous cycle [37]. The ampulla possesses a thicker muscularis and the mucosal projections are shorter than in the infundibulum. Some of them do not tend to unite with each other (Fig 3A and 3B). The lining epithelium is like the one of the infundibulum (Fig 3C). In accordance with previous descriptions in the cow [35], at the isthmus, the muscularis reaches its maximum thickness and the epithelial extensions its minimum length (Fig 3D and 3E). The epithelium in the isthmus is like the one of the infundibulum and the ampulla (Fig 3F).

### Uterus

**Fig 4:**
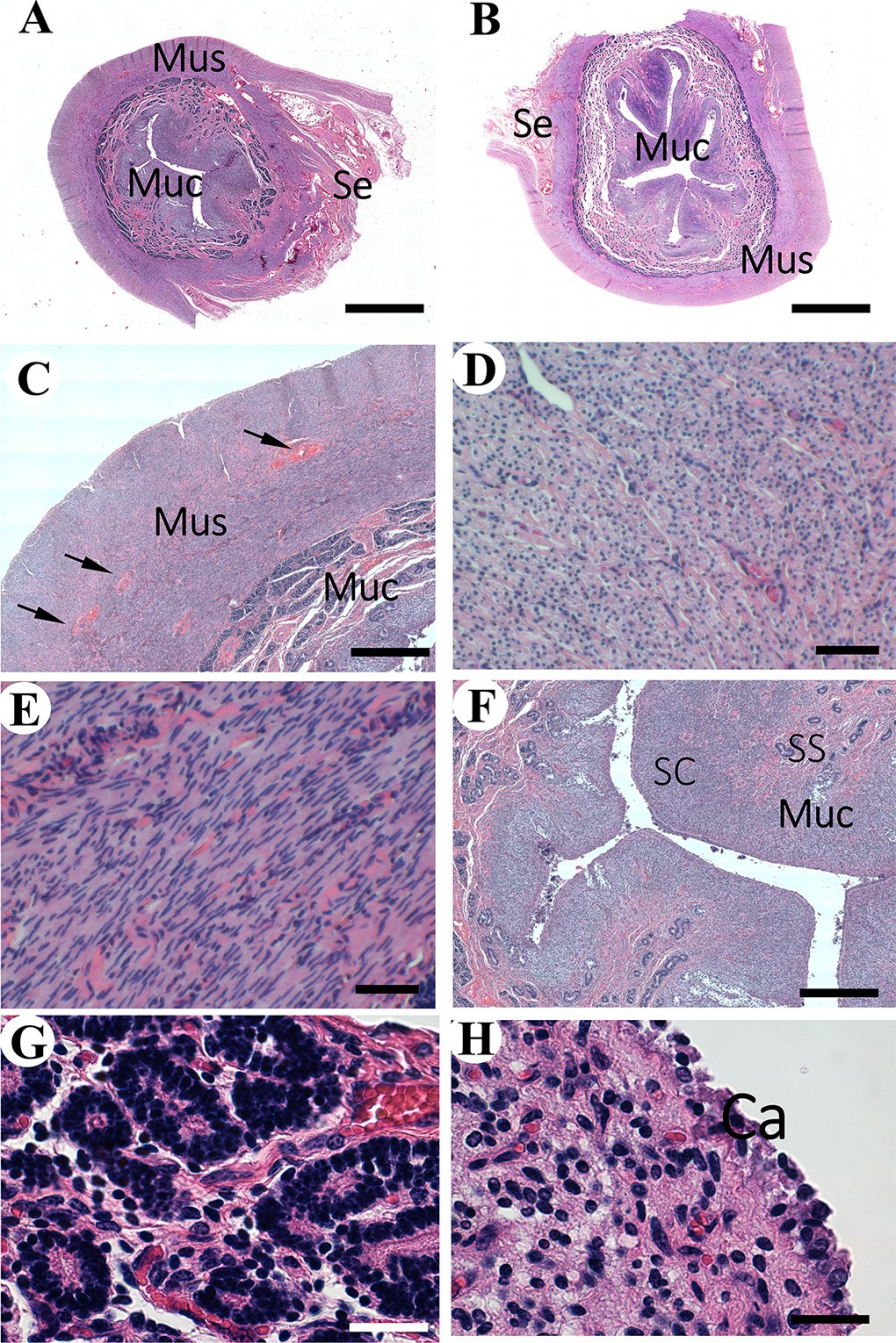
Histology of the uterine horns of the Arabian oryx. A) Overview of the transversal section of the proximal region. Serosa (Se), muscularis (Mus), and mucosa (Muc) (Bar = 1 mm). B) Overview of the transversal section of the distal region. Note the similarity with A (Bar = 1 mm). C) Magnification of Mus and Muc of A. Blood vessels of straum vasculare (arrows) located beetwen the external and internal layers of smooth muscle (Bar = 400 μm). D) External layer of Mus. Note the transversal sections of the cells, indicating that the fibers are located in a longitudinal fashion (Bar = 50 μm). E) Internal layer of Mus. Note the longitudinal sections of the cells, indicating that the fibers are located in a circular orientation (Bar = 50 μm). F) Magnification of Muc, stratum compactum (SC) and stratum spengiosus (SS) (Bar = 500 μm). G) Uterine glands in the lamina propria of Muc (Bar = 25 μm). H) Magnification of the caruncles (Bar = 25 μm).

The uterus provides space and nutrition for the developing embryo and fetus and expulses the latter at birth. Unlike domestic ruminants, the oryx uterus has no distinctive body and each uterine horn opens independently into a cervical canal [21]. Despite this notable difference, our study indicates that the microstructure of the uterine walls is the same as in other species, particularly ruminants [22, 24]. The proximal and distal regions of the uterine horns possess the same structure, with minor differences in the morphology of the mucosa. The uterine wall is made of three layers: serosa (perimetrium), muscularis (myometrium), and mucosa (endometrium) (Fig 4A and 4B). The perimetrium has the typical serous membrane, made of simple cuboidal cells. Like in the cow and other species [22, 24], the myometrium is composed of two muscular layers separated by a thick layer of connective tissue that is rich in blood vessels (stratum vasculare) (Fig 4C). The thinner layer, located externally to the stratum vasculare, is made of fibers arranged longitudinally (Fig 4D). In contrast, the internal layer is made of fibers with a circular orientation (Fig 4E). The endometrium is highly cellular with numerous capillaries and like in the cow [38], it is organized into two zones. The deeper one is the stratum spongiosum, while the superficial one is the stratum compactum. The stratum spongiosum is made of connective tissue that embeds numerous glands which are coiled and lined by a simple cuboidal epithelium (Fig 4F and 4G). The stratum spongiosum extends into the superficial zone, the stratum compactum. This stratum is lined by a simple columnar epithelium and possesses wide and round regions of connective tissue that extend into the lumen. These are the caruncles (Fig 4H). They provide attachment for the fetal membranes and are characteristic of ruminants [24, 39].

### Cervix

**Fig 5:**
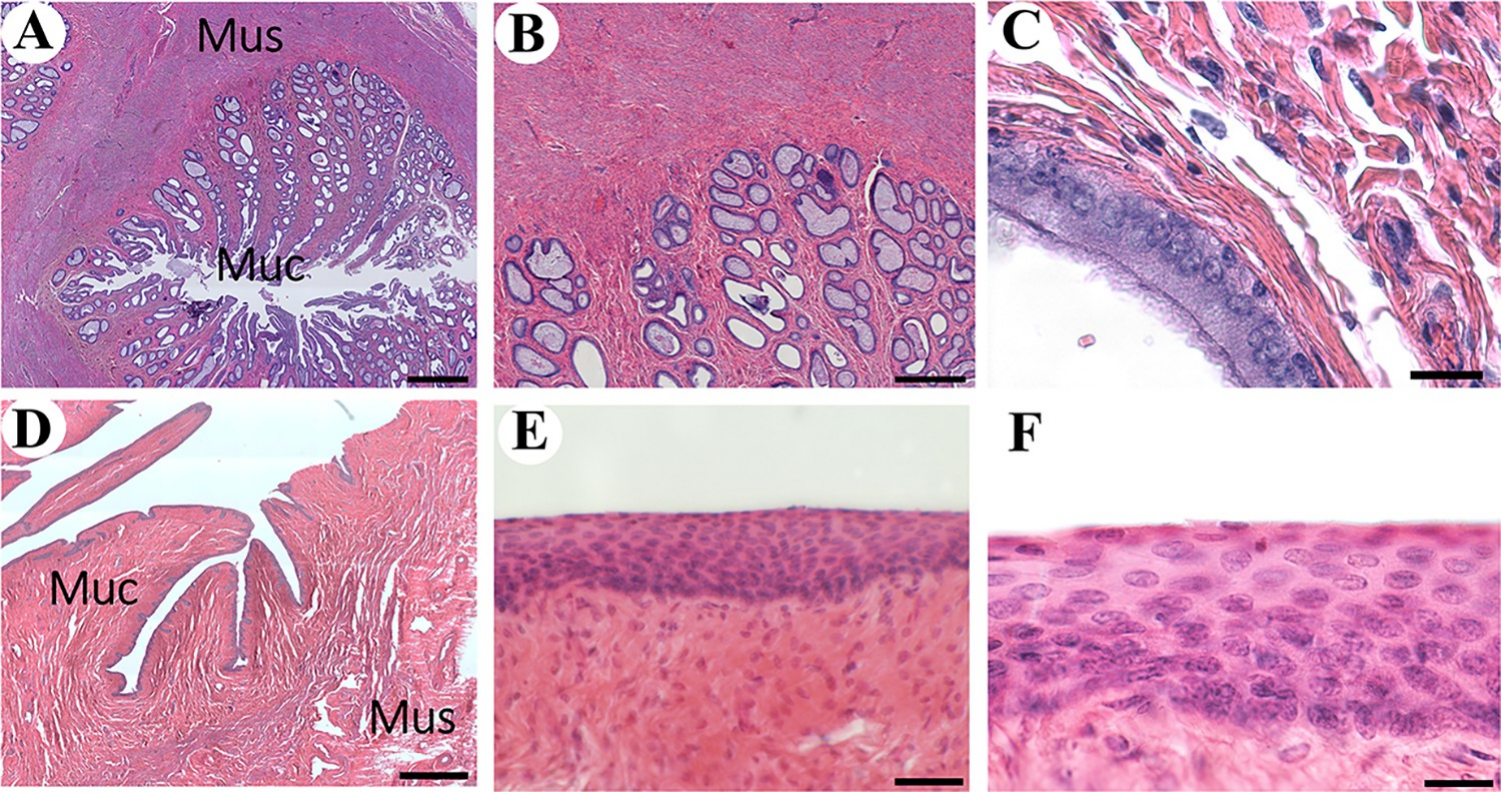
Histology of the cervix and vagina of the Arabian oryx. A) Overview of the transversal section of the right cervical canal. Mucosa (Muc) and Muscularis (Mus) (Bar = 1 mm). B) Magnification of Muc. Note the presence of numerous glands (Bar = 400 μm). C) Epithelial lining of Muc, columnar epithelium with ciliated cells (Bar = 20 μm). D) Overview of transerval section of the cranial region of the vagina (Bar = 1 mm). E) Epithelial lining of Muc, made of stratified squamous epithelium (Bar = 50 μm). F) Magnifcation of E (Bar = 20 μm).

As mentioned above, unlike the cow [22], the cervix of the Arabian oryx possesses two canals, one for each uterine horn [21]. No difference was noted in the microstructure of both canals. Like the uterus, the cervix is made of serosa, muscularis, and mucosa. The serosa is an extension of the one of the uterus. The muscularis is made of a thick smooth muscle, which is a continuation of the one of the uterine walls (Fig 5A). The canals are lined by a mucosa that extends into the lumen as longitudinal irregular folds. At the base of the folds, where they tend to merge, there are numerous large mucus glands that form a thick layer (Fig 5B). The glandular epithelium is cuboidal and columnar cells. A simple columnar epithelium with ciliated cells covers the border of the folds at the non-glandular region (Fig 5C). Despite having two independent canals, this microstructure is similar to the one described in the cow and other species [24].

### Vagina

In the present study, we could only evaluate the cranial region of the vagina due to the rest was not provided with the other organs. The walls of this are constituted by a mucosa, muscularis, and serosa (Fig 5D). The serosa and muscularis are extensions of the ones of the cervix. A stratified squamous non-keratinized epithelium lines the mucosa (Fig 5E and 5F). In the cow this epithelium in this region has been described as stratified columnar [24]. This difference needs farther clarification by additional studies analyzing more specimens.

## Conclusion

We studied the histology of the internal reproductive organs of one female Arabian oryx and found consistent features with those described in other mammals, especially ruminants. This indicates that, despite having studied a single specimen, the histology of the internal reproductive organs of the female Arabian oryx and ruminants is similar. The reason for the difference found in the epithelium of the cranial region of the vagina of the Arabian oryx and that of the cow is uncertain. To determine if this is an individual or species variation is necessary to study more specimens. This will also confirm our results and detect specific individual variations. In the best of our knowledge, this is the first full report on the reproductive histology in the Arabian oryx and other species of antelopes. It will contribute to understanding the reproductive function of these species and developing strategies for their conservation.
